# Thyroid function and opium use disorder: a cross-sectional study on the Fasa adults cohort study (FACS), 2017

**DOI:** 10.1186/s12902-023-01467-3

**Published:** 2023-11-30

**Authors:** Babak Pezeshki, Hossein Pourmontaseri, Reza Homayounfar, Maryam Talebi Moghaddam, Azizallah Dehghan

**Affiliations:** 1 Noncommunicable Diseases Research Center, Fasa University of Medical Sciences, Fasa, Iran; 2 Student Research Committee, Fasa University of Medical Sciences, Fasa, Iran; 3 USERN Office, Shiraz University of Medical Sciences, Shiraz, Iran; 4 Shiraz Nutrition Interest Group, Bitab Enterprise, Shiraz, Iran; 5 Faculty of Nutrition Sciences and Food Technology, National Nutrition and Food Technology Research Institute (WHO Collaborating Center), Shahid Beheshti University of Medical Sciences, Tehran, Iran; 6 Noncommunicable Diseases Research Center, Fasa University of Medical Sciences, Fasa, Iran; 7 Department of Epidemiology and Biostatistics, School of Public Health, Kerman University of Medical Sciences, Kerman, Iran

**Keywords:** Addiction, Thyroid hormones, Opioids, Risk factors

## Abstract

**Background:**

Addiction increases the risk of different lifelong disorders. However, there are limited studies evaluating the effects of opioid use disorder (OUD) on thyroid function. The present study aimed to compare the thyroid function of individuals with and without OUD.

**Methods:**

This cross-sectional study was conducted on 700 eligible participants of the Persian Cohort of Fasa, Iran. Pregnant women and participants with false or missing data were excluded from the study. Remained participants were divided into case and control groups based on the recorded history of OUD. Frozen plasma samples of the cohort bank were used to determine the levels of T3, T4, and thyroid-stimulating hormone (TSH). The thyroid function was compared between the two groups using the Mann-Whitney test (P < 0.05).

**Results:**

The mean age of the final studied population (n = 648) was 54.0 ± 9.8 years, including 336 men (49.1%) and 197 participants with OUD (28.8%). The median levels of TSH, T4, and T3 were 2.91 ± 4.61, 9.26 ± 3.65, and 1.22 ± 0.49, respectively. The case group had significantly higher TSH (3.72 ± 6.2 vs. 2.58 ± 3.75, P < 0.001) and lower T4 (8 ± 3.6 vs. 9.8 ± 3.5, P < 0.001). Also, T3 was slightly lower in the case group (1.1 ± 0.5 vs. 1.3 ± 0.5; P = 0.369), although this association was only significant in female opium users (P < 0.001).

**Conclusions:**

The present findings revealed that OUD caused a reduction in T4 while increasing TSH. Therefore, OUD may lead to the development of primary hypothyroidism, which needs to be investigated in future studies.

## Introduction

Thyroid hormones (THs) play several important roles in the development of metabolism and growth [1, 2]. TH dysregulation causes a group of disorders, including hypothyroidism and hyperthyroidism [3]. Over the past decades, several tools and therapies have been developed to control THs disorders worldwide [4, 5]. Although TH disorders can be diagnosed and treated effectively, global reports suggest that more reliable approaches to the addictions and individual habits of patients are needed [6, 7]. Besides, the prevalence of TH disorders is growing worldwide (0.2–1.3% in some regions) [8, 9], and Iranian reports of iodization have indicated the concerning incidence of hypothyroidism (328 per 100,000) and hyperthyroidism (88 per 100,000) in various regions [10].

For many years, the hypothalamic-pituitary (HP) axis has been known to regulate the level of THs by releasing thyroid-stimulating hormone (TSH); this axis has a close association with TH functions in different types of metabolism [11]. According to numerous trials, addiction to various substances results in the HP axis dysfunction and causes multiple disorders [12–14]. While some studies indicated that substance abuse caused a significant downregulation of HP axis, the others revealed that this association was insignificant [15–17].

In the past decades, abuse of narcotic substances has globally increased [18]. Recently, the World Health Organization (WHO) reported that among Islamic countries, Iran has the highest rate of opioids abuse [19]. Animal studies suggest that consumption of narcotics results in thyroid dysfunction [20, 21]. Besides, in some trials, diminished T3 uptake has been shown in opium abusers [22]. Therefore, since existing evidence reveals that OUD would result in significant thyroid dysfunction, further studies are required to address this association [23].

With this background in mind, since no similar study has been conducted in Fasa University of Medical Sciences (Fars Province, Iran), the present study aimed to investigate the association between OUD and TH levels (T3, T4, and TSH).

## Materials and methods

### Study design

This cross-sectional study was conducted on the participants of the Fasa Adults Cohort Study (FACS). In this prospective Cohort study, the individuals aged 35 to 70 years were collected from Sheshdeh, Fasa, Iran (n = 10,118). In 2016, three detailed Questionnaires, including baseline features, medical history, and anthropometrics, were fulfilled by experienced examiners from participants to investigate prevalence, etiologies, risk factors, mechanisms, comorbidities and incidence of chronic noncommunicable diseases [24].

Considering the power of 90%, type 1 error of 0.05, and according to the study of Shahesvari et al., the minimum sample size for the study was calculated to be 90 people in each group.; the participants were randomly recruited in the study [22]. All of the participants with stored plasma samples and recorded related data were included in the study. Initially, 700 participants were simple randomly selected. The participants with incomplete data or missing samples were excluded. Also, participants with false data (if the urine test result did not match the patient’s self-report) were eliminated from the study.

### Measurements

The procedure of data collection is described comprehensively in the protocol of the FACS [25]. The OUD group was defined as the individuals who reported a history of using each category of natural or synthetic injected, inhaled, or oral opioids (e.g. opium, oxycodone, methadone, morphine, and tramadol). To confirm the validity of the records, the examiners of FACS evaluated the appearance of withdrawal symptoms and history of opioid consumptions, based of ICD-10 guideline [26].

The plasma samples of the selected participants were used to measure the levels of THs using the ELISA technique. The normal ranges of T3, T4, and TSH were as follows: 0.52–1.85 ng/mL, 4.8–14.8 ug/dL, and 0.3–6.1 mIU/L, respectively.

### Ethical considerations

The present results were extracted from a research project, approved by Fasa University of Medical Sciences, with an ethics code of IR.FUMS.REC.1399.094. In line with ethical and confidentiality principles, the objectives and principles of the research, confidentiality of data, and anonymity of the questionnaire were explained to all the participants. They were also free to refuse participation in the study at any time. All included participants completed informed written consent before participation in the present study.

### Statistical analysis

Descriptive data were reported as mean ± standard deviation (SD) and frequency. Data were analyzed in SPSS version 16, using t-test and Mann-Whitney U test. Kolmogorov-Smirnov test was also applied to evaluate the normal distribution of variables. The level of each hormone was compared between the user and non-user groups. The participants were also divided into three groups, based on the normal range of each hormone. To investigate the association of opium use with the level of each hormone, multinomial logistic regression was performed. The significance level was set at P < 0.05.

## Results

A total of 684 participants were evaluated in the present study. The mean age of the population, including 336 (49.1%) men, was 54.0 ± 9.8 years. Overall, 28.8% of the participants (n = 197) were opium users. In this population, there were 25 female opium users, which is significantly lower than the number of men (P < 0.001). Based on the results, the rate of obesity was significantly lower in opium users (P = 0.006). On the other hand, myocardial infarction was more frequent in opium users (P = 0.002). There was no significant difference between the groups regarding age, diabetes, cardiovascular disease, and TH disorders (Table 1).

**Table 1:** Characteristics and comparison of demographic features between opium users and non-users

variable	subgroup	User (N = 197) Mean ± SD or frequency (percent)	Nonuser (n = 487) Mean ± SD or frequency (percent)	p-value*
gender	men	162(82.2)	174(35.7)	**0.001**
	women	35(17.8)	313(64.3)	
age		53.61 ± 9.33	54.12 ± 10.0	**0.539**
BMI	normal	98(49.7)	178(36.6)	**0.006**
	Over weight	67(34)	212(43.5)	
	obese	32(16.2)	97(19.9)	
Diabetes	yes	57(28.9)	145(29.8)	**0.827**
	no	140(71.1)	342(70.2)	
Cardiac	yes	89(45.2)	197(40.5)	**0.256**
	no	108(54.8)	290(59.5)	
MI	yes	56(28.4)	87(17.9)	**0.002**
	no	141(71.6)	400(82.1)	

* The significant level of independent T-test and Chi-square test was considered P-value < 0.05

The level of TSH was dramatically higher in OUD group (P < 0.001). The subgroup analysis showed that this difference was insignificant in men (P = 0.462) and significant in women (P = 0.019). However, all other classifications (shown in Table 2) revealed no remarkable differences, except obese participants (P = 0.011).

**Table 2:** Comparision of thyroid related hormones between opium users and non-users

Variable	Subgroup	TSH			T3			T4		
		User (n = 197) Mean ± SD	Nonuser (n = 487) Mean ± SD	p-value*	User (n = 197) Mean ± SD	Nonuser (n = 487) Mean ± SD	**p-value**	User (n = 197) Mean ± SD	Nonuser (n = 487) Mean ± SD	**p-value**
	Total	3.72 ± 6.2	2.58 ± 3.75	**0.001**	1.1 ± 0.5	1.3 ± 0.5	**0.369**	8 ± 3.6	9.8 ± 3.5	**0.001**
Gender	men	3.31 ± 5.85	2.56 ± 3.81	**0.462**	1.15 ± 0.46	1.27 ± 0.45	**0.068**	8.62 ± 3.61	9.65 ± 3.68	**0.018**
	women	5.6 ± 7.41	2.60 ± 3.72	**0.019**	0.72 ± 0.46	1.27 ± 0.50	**0.001**	5.36 ± 2.41	9.82 ± 3.45	**0.001**
Age	Q1	3.59 ± 5.75	2.90 ± 5.11	**0.179**	1.10 ± 0.51	1.38 ± 0.57	**0.003**	7.93 ± 3.72	10.13 ± 3.85	**0.001**
	Q2	3.16 ± 4.69	2.15 ± 1.55	**0.957**	1.04 ± 0.44	1.23 ± 0.45	**0.034**	8.21 ± 3.55	9.50 ± 3.32	**0.024**
	Q3	4.14 ± 7.16	2.59 ± 3.57	**0.369**	1.08 ± 0.48	1.18 ± 0.42	**0.169**	8.27 ± 3.26	9.60 ± 3.65	**0.063**
	Q4	4.01 ± 7.11	2.56 ± 3.29	**0.453**	1.07 ± 0.54	1.27 ± 0.45	**0.053**	7.7 ± 4.17	9.69 ± 3.19	**0.001**
Body mass index	normal	3.35 ± 4.91	2.46 ± 3.37	**0.092**	1.17 ± 0.47	1.30 ± 0.51	**0.156**	8.36 ± 3.74	9.97 ± 3.73	**0.002**
	Over weight	4.71 ± 8.14	2.51 ± 4.42	**0.106**	0.93 ± 0.49	1.27 ± 0.48	**0.001**	7.4 ± 3.61	9.63 ± 3.48	**0.001**
	obese	2.78 ± 4.82	2.96 ± 2.66	**0.011**	1.07 ± 0.47	1.23 ± 0.43	**0.116**	8.40 ± 3.29	9.64 ± 3.28	**0.108**
Diabetes	yes	4.58 ± 7.36	2.29 ± 2.09	**0.915**	1.04 ± 0.53	1.28 ± 0.46	**0.003**	8.10 ± 4.14	9.28 ± 2.99	**0.023**
	no	3.37 ± 5.65	2.71 ± 4.26	**0.321**	1.08 ± 0.47	1.26 ± 0.49	**0.001**	8.01 ± 3.43	9.96 ± 3.73	**0.001**
Cardiac	yes	3.64 ± 5.66	2.61 ± 4.40	**0.718**	1.1 ± 0.49	1.31 ± 0.52	**0.012**	8.29 ± 3.72	10.14 ± 3.65	**0.001**
	no	3.78 ± 6.64	2.56 ± 3.24	**0.409**	1.04 ± 0.49	1.25 ± 0.46	**0.001**	7.83 ± 3.58	9.49 ± 3.44	**0.001**
Myocardial infarction	yes	2.66 ± 3.98	2.15 ± 1.78	**0.483**	1.2 ± 0.44	1.20 ± 0.45	**0.511**	8.52 ± 3.33	9.69 ± 3.05	**0.035**
	no	4.14 ± 6.85	2.67 ± 4.05	**0.149**	1.02 ± 0.50	1.29 ± 0.49	**0.001**	7.85 ± 3.75	9.77 ± 3.63	**0.001**

* The significant difference of thyroid related hormones between users and non-users was considered P-value < 0.05 (Mann-Whitney U tests)

The level of T4 was significantly lower in OUD group compared to non-users (P < 0.001), and the difference remained significant in both sexes, body mass index subgroups, and all age quartiles. Also, after dividing the participants into healthy and participants with a history of chronic diseases, the difference remained significant (Table 2). Conversely, the difference in T3 level between case and control groups was insignificant, although OUD group had slightly lower levels of T3 (P = 0.369). However, the difference was significant in women (P < 0.001) and nearly significant in men (P = 0.068); this association became significant in the age subgroups. Although T3 was dramatically lower in overweight participants, the difference remained insignificant in other subgroups. The level of T3 was statistically equal in individuals with a history of myocardial infarction, whereas participants with or without diabetes and cardiac disease showed significant differences‌ (Table 2).

Table 3 presents the frequency of OUD and non-OUD participants in each group of thyroid function, based on the normal ranges of TSH, T4, and T3. Importantly, the present findings revealed that all participants with low levels of T3 and T4 were participants with OUD. Also, less than 20% of participants with high levels of T3 and T4 had OUD. Table 3 indicates that case group had significantly lower levels of T3 and T4. The multinomial regression analysis showed that case group had a significantly lower risk of high T4 levels (OR, 0.74; 95% CI: 0.33–1.68); this association was insignificant regarding T3 levels (OR, 0.71; 95% CI: 0.31–1.60).

**Table 3:** The association of opium consumption with different level of thyroid related hormones

Group	Non-user	User	Chi-square P value*	crude model OR (95%CI) **	Adjusted model OR (95%CI) ***
low-TSH	30	8	0.012	0.69 (0.31, 1.55)	0.67 (0.26, 1.71)
Normal-TSH	427	164		1 (ref)	1 (ref)
High-TSH	30	25		2.17 (1.24, 3.80)	3.21 (1.66, 6.22)
low_T4	0	55	< 0.001	ERROR	ERROR
Normal_T4	431	130		1 (ref)	1 (ref)
high_T4	56	12		0.71 (0.37, 1.37)	0.74 (0.33, 1.68)
low_T3	0	38	< 0.001	ERROR	ERROR
Normal_T3	436	146		1 (ref)	1 (ref)
high_T3	51	13		0.76 (0.40, 1.44)	0.71 (0.31, 1.60)

* The significant difference of thyroid related hormones between users and non-users was considered P-value < 0.05 (Chi-square)

** The crude model was performed adjusted for no covariates

*** The adjusted model was performed adjusted for gender + age + body mass index + educational status + smoking + alcohol consumption + diabetes + myocardial infarction + cardiac disease + hypertension + fatty liver disease

Regarding TSH levels, a reverse association was observed. The OUD only accounted for 21.1% of cases with low TSH levels, while 45% of them had high levels of TSH; this finding suggests that OUD is associated with higher TSH levels. The unadjusted regression model showed that case group had a significantly greater risk of high TSH levels (OR, 2.17; 95% CI: 1.24–3.80); this association became more prominent after adjustment (OR, 3.21; 95% CI: 1.66–6.22). Therefore, according to this model, OUD had a close independent association with high TSH levels. In other words, opium users had a lower chance of low TSH levels (OR, 0.67; 95% CI: 0.26–1.71).

## Discussion

This study aimed to evaluate the association between OUD and TH levels (T3, T4, and TSH). The findings showed that T3 and T4 levels decreased in participants with a history of OUD, which might suggest hypothyroidism. On the other hand, OUD was associated with higher TSH levels. Overall, it is speculated that chronic dependency to opioids is associated with primary hypothyroidism.

Some studies have shown that alcohol, cocaine, and heroin addiction have no significant effects on the secretion of pituitary hormones and possibly, T3 and T4 levels. However, others have indicated changes in T3 and T4 levels, mostly due to the direct effects of these substances on the thyroid gland [27, 28]. A study by Dogar et al. reported controversial results regarding the inhibitory effects of opium on TH levels. This observation showed that addicts had higher T3 levels compared to healthy individuals [29]. Rasheed et al. also found that heroin abuse increased T3 levels compared to the control group. Additionally, the level of T4 was not significantly different between addict and non-addict groups [15].

In another study by Brambilla et al., the results showed that addictive substances had no effects on T3 and T4 levels [30]. Although some studies have emphasized the effects of these addictive substances on lowering THs, some studies show no TSH-lowering effects. It should be noted that differences in sample size, type of addictive substances, and studied populations might result in these controversies. Nonetheless, it can be concluded that these substances have some suppressive or destructive effects on the pituitary gland, which are associated with secondary hypothyroidism pathogenesis.

The hypothalamic-pituitary-thyroid axis is a continuous longitudinal pathway, which involves several biological mechanisms. Negative feedback increases the level of TSH, followed by a reduction in T4 and T3 levels [31]. The present study revealed that opium simultaneously suppressed the thyroid gland function and induced the activity of pituitary hormones. Theoretically, it can be concluded that opium use is associated with hypothyroidism (probably primary hypothyroidism), which is followed by a rise in TSH levels. In a study by Gozashti et al., comparing the thyroid function of 50 addict and 50 non-addict participants, similar findings were reported, although our study concentrated on the distribution of THs that influenced the reliability of results [23]. Basically, THs are not normally distributed, even in large sample sizes [32]. Therefore, it is important to apply nonparametric tests to investigate different aspects of THs.

The present study showed that TSH, T3, and T4 levels had a significant non-normal distribution. Consequently, Mann-Whitney U test was applied to compare the levels of these hormones between opium users and non-users. Nonetheless, normal distribution is an important assumption in linear regressions; therefore, we could not perform this analysis. In the current study, the participants were divided into low, normal, and high hormone levels, based on the normal range of each hormone. Next, multinomial regression analysis was performed to investigate the risk of each pathological condition in opium users. Finally, our findings supported that opium users had a significantly higher chance of lower T4 and T3 levels and higher TSH.

The results of studies by Zhang et al. [33] and Bhoir et al. [34] are consistent with ours. Although the correlation of opium use with hypothyroidism was significant in some previous studies, no significant difference was found relative to the control group. According to previous observations, lower TH levels are correlated with the level of thyroxine-binding globulin (TBG) in the liver tissue. Generally, the T3 resin uptake (T3RU) assay is the most commonly applied tool for determining TBG levels [35]. Accordingly, the overall profile of T3 and T4 hormones should be evaluated to make more accurate inferences from the results.

A study by Shenkman et al. reported that TSH levels were normal in addicts compared to the control group [36]. Also, Brambilla et al. showed that heroin abuse did not affect TSH levels [30]. Some studies have not examined TSH levels alongside other hormones [29]. In line with the present study, Pende et al. documented increased TSH levels following buprenorphine and heroin abuse [37].

Different theories have hypothesized the downregulation of T4 and T3 in opium users. Since our results suggest the significant risk of primary hypothyroidism, opium use can induce cellular death or suppression in the thyroid glands. A review study by Mirzaei et al. emphasized that opium and its derivatives induce apoptosis in different cells, especially in the liver, brain, and immune system [38]. Remarkably, opium upregulated both extrinsic and intrinsic pathways of apoptosis through activation of caspase 9, caspase 8, and caspase 3 [39–41]. The probable destructive effects of chronic opium abuse on thyroid cells should be investigated in future studies.

The present study had some limitations. Regarding the common characteristics of cross-sectional studies, our findings showed no cause-and-effect association between opium use and TH levels. On the other hand, for legal and ethical reasons, it was not possible to conduct interventional studies to determine the effect of opium on thyroid function; therefore, further retrospective and prospective cohort studies are required to overcome this limitation. Besides, there are still some controversies about TSH levels in opium users. The current findings support the possibility of primary hypothyroidism and the indirect effect of opium use on the pituitary gland, based on negative feedback. Nevertheless, further studies are required to investigate the molecular and cellular mechanisms of this condition.

The present study had several strengths. First, the large sample size of the Persian Cohort of Fasa, which was used in the present study for the first time, provided some new information. Second, our clinical approach of dividing the participants into low, normal, and high levels of each hormone provided more reliable results. Third, the patients were classified into different categories of thyroid function, and their findings were compared with those of a normal population (separately for each hormone). However, future studies with a larger sample size are required to investigate the odds ratio of each pathological condition in opium users.

## Conclusion

According to the findings of the current study, opium abuse can influence the thyroid function by affecting the levels of T3 and T4 hormones. The results also suggested that opium use led to primary hypothyroidism through destruction or suppression of the thyroid gland. Future studies are suggested on a larger sample size with sex-related adjustments and free TH measurements.

## Ethics approval and consent to participate

The present study was conducted based on Helsinki Declaration. The results of this study have been adapted from a research project approved by Fasa University of Medical Sciences with a code of ethics of IR.FUMS.REC.1399.094 In line with the principles of ethics and confidentiality, all participants in the study were explained about the principles and objectives of the research, the confidentiality of the data and the anonymity of the questionnaire. In addition, they were free to refuse to participate. Also, the certification of ethics approval is uploaded as related file. The Ethics committee of Fasa university of medical science had the high standard observation and validation of informed consent of all eligible individuals which were participated in the present study. The PERSIAN Cohort of Fasa had an approved protocol which were confirmed by Ethics committee of Fasa university of medical science and published in PubMed [42]

## Data Availability

The data which was analyzed in the present study will be available for the researchers who ask the corresponding author to share them the dataset with ethical approval. In contrast, the Noncommunicable Diseases Research Center of Fasa University of Medical Sciences asked all researchers to respect data confidentiality. As a result, the permission to make the database available depends on having the permission of the Ethics Committee of Fasa University of Medical Sciences.
